# Cohesin as an essential disruptor of chromosome organization

**DOI:** 10.1016/j.molcel.2025.01.010

**Published:** 2025-02-04

**Authors:** Adrian Bird

**Affiliations:** 1 Wellcome Centre for Cell Biology, University of Edinburgh, The Michael Swann Building, Edinburgh EH9 3BF, UK

## Abstract

Cohesin is a multi-subunit molecular machine that is able to create lateral chromatin loops within a linear chromosome fiber. Despite intense study, a consensus view of the functional significance of loop extrusion has remained elusive. This perspective proposes a rationale based on the need for continual disruption of spurious higher-order chromatin secondary structures. It is argued that cohesin-mediated chromosomal churn ensures broad accessibility to the diffusible factors on which genome function depends.

## Chromosomal Loops and Domains

Each eukaryotic chromosome consists of a single DNA duplex that extends uninterrupted from one end to the other, capped by telomeres. Despite DNA continuity, it has long been suspected that functional subdivisions are imposed on the DNA via chromatin-associated proteins. A prominent current view is that the genome is organized as a series of dynamic loops within domains that are often bordered by insulating proteins (e.g., tightly bound CTCF protein dimers). Responsible for this predominantly segmented structure is a protein complex called cohesin, which is capable of extruding loops *de novo* from linear DNA.^1^ The aftermath of this ATP-dependent process can be visualized experimentally as “topologically associated domains” (TADs) within which intra-chromosomal contacts are preferred over contacts with chromosomal regions beyond the TAD boundaries.^2–4^ Thus, TADs may represent domains within which interactions between a gene and its cognate regulatory regions are favored. A variant of this view is that the process by which TADs form facilitates enhancer-promoter interactions by improving the likelihood of encounters between regulatory DNA sequences that may rarely collide by chance (for potential mechanistic models, see Mach et al.^5^ and Xiao et al.^6^). This article emphasizes an alternative functional perspective on cohesin-mediated looping by arguing that the primary role of extrusion may be to achieve disruption of nascent chromatin aggregates to ensure continuous access for diffusing nuclear proteins and complexes. Rather than creating units of chromosome organization, this view would suggest that in most cases TADs are the detectable shadow of essential genomic churn.

The notion that loops underlie chromosome organization goes back to the observation of “lampbrush chromosomes” in big-genomed amphibia.^7^ Each meiotic bivalent could be seen in the light microscope to consist of a string of heterogeneous bead-like structures from which emanated pairs of loops. An apparently comparable structure was later seen in mammalian mitotic chromosomes following the removal of all histones.^8^ Organization into loops was not therefore peculiar to amphibian meiosis but could be a consistent feature of all eukaryotic chromosomes. Support for this notion came with the demonstration that the cohesin complex, known initially for its role in keeping sister chromatids together after DNA replication, can itself extrude DNA loops, as had been speculated previously.^9,10^ TADs, as detected by Hi-C and related methods, provide a visible manifestation of this process. These discoveries fed the perception that TADs define a tandem series of territories along the chromosome, each of which functions as a self-contained unit of gene regulation (for a more comprehensive historical account, see Yatskevich et al.^11^). By physically confining enhancer-promoter interactions to a single territory while excluding contacts with DNA elsewhere, TADs would not merely organize the genome but would be crucial determinants of gene activity.

### Is Cohesin a Builder or Destroyer Of Chromosomal Interactions?

With the passage of time, experimental work has progressively challenged this hypothesis. Firstly, rather than being fixtures of chromosome structure, loops prove to be transient. In fact, they are absent at least half the time at any specific genomic locus, as measured by the association frequency of boundary CTCF sites.^5,12^ Secondly, and probably related, the insulation provided by TADs is weak, as the preference for contacts within a TAD is only ∼2-fold greater than between neighboring TADS.^12^ Dissolution of TADs has a small effect on the efficiency of most promoter-enhancer interactions, indicating that looping is not necessary to prevent interference from remote enhancers.^13,14^ Thirdly, cohesin and TADs appear to have a limited role in gene regulation, as acute TAD ablation minimally disrupts normal gene expression.^13–17^ Correct activity of certain genes undoubtedly involves cohesin (e.g., in directing variable-diversity-joining recombination in the immune system^18^ and the regulation of cadherin gene expression^19^), but direct involvement of cohesin action in regulating gene expression appears to be limited rather than general.

**Figure 1:**
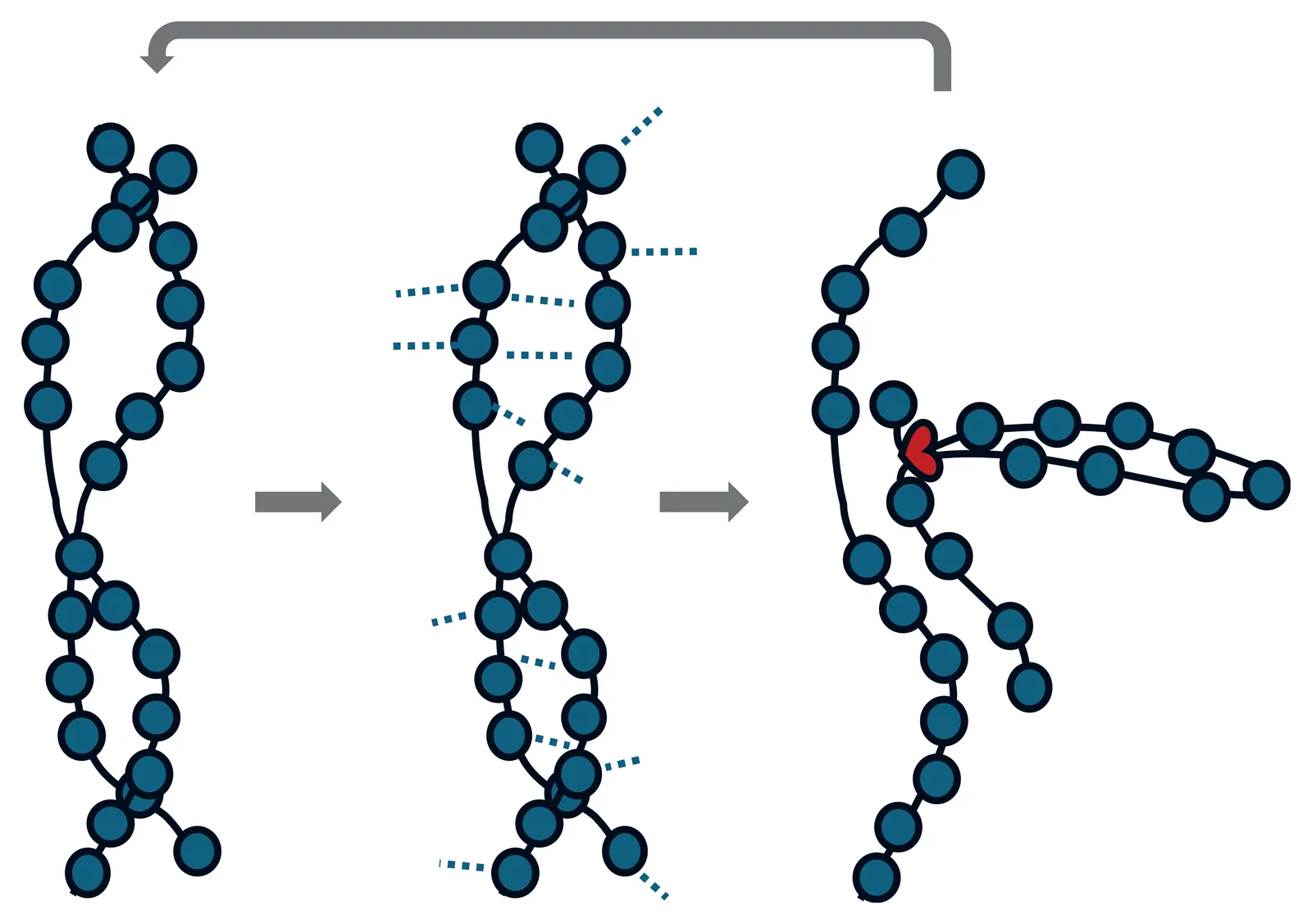
Cartoon representing two DNA strands in close proximity (black lines) plus associated histones and other proteins (blue circles) Spurious secondary interactions develop over time (dotted lines) but are periodically disrupted by feeding chromatin through cohesin to form transient loops.

Contrasting with the constructive vision of cohesin function is the possibility that the primary role of loop extrusion is disruptive. In the absence of continual mixing, it can be argued, the genome tends to “congeal,” thereby restricting access to proteins and protein complexes that mediate essential genome activities, including transcription, replication, and repair of DNA. Repeated cycles of extrusion are needed to ensure that over time chromatin is maximally exposed to the influence of proteins in the nuclear environment. In this way, looping may solve a biophysical problem inherent to the extended polymeric nature of the genetic material, exacerbated by its chemically diverse macromolecular coating. Without mixing, chromatin threads may form lateral contacts due to multiple weak, non-specific, or otherwise inappropriate macromolecular contacts (Figure 1). A potential example of such a tendency is provided by the pre-mitotic compaction of chromosomes induced by histone deacetylation.^20^ Extrusion would work against such “ossification” by tearing spurious secondary structures apart while creating loops as an incidental by-product. This might help to explain why DNA often spends more time unlooped than looped. The purpose of the exercise is not to make loops but to break nascent chromatin associations and refresh DNA availability, a process that only needs to be repeated every few minutes. Rather than guiding intragenomic contacts through DNA spooling, loop extrusion would be simply permissive, giving free rein to molecular diffusion of factors, encouraging unfettered mass action.

### The Importance of Genomic Churn

There is already evidence for disruptive churn of this kind and some support for its functional importance. Based on computer simulations, it has been proposed that “normal” chromatin, comprising irregularly folded nucleosomal chromatin fibers (10 nm fibers), is impenetrable to much macromolecular machinery,^21^ implying that special measures are needed to open it up. Indeed, occupation of promoters and enhancers by transcription factors is significantly reduced after sudden loss of cohesin or CTCF, as expected if availability of target DNA sequence motifs is compromised.^14^ The authors suggest that this reveals an “underappreciated aspect of cohesin function.” Several further studies have confirmed the disruptive effects of cohesin activity. Predictions from polymer modeling that loop extrusion overrides the tendency of chromosomes to segregate into compartments^22^ were confirmed by the observation that high levels of cohesin actively counteract genome compartmentalization.^23^ It followed that loop extrusion by cohesin acts as a “mixing device, stirring up chromatin and breaking up interactions that tend to form between genomic regions sharing the same chromatin modifications.”^11^ Related logic may apply in prokaryotes, where cohesin-related proteins contribute to chromosome segregation at cell division. The mere disruption of spurious higher-order interactions may be sufficient to ensure the necessary “individualization” of daughter genomes.^24^ It is conceivable, therefore, that the destructive activity of cohesin-like proteins corresponds to their original function, shared by all life forms. Other roles, including insulation, facilitation of promoter-enhancer contacts, and even sister chromatid cohesion, may have evolved secondarily.

An impressive illustration of the consequences for gene expression is provided by the cohesin-dependent disruption of long-range interactions between polycomb-repressed genes.^25^ The interpretation is that aggregation of different repressed loci is an unintended consequence of silencing by polycomb repressive complex 1 (PRC1) that can lead to spurious hyper-repression unless it is repeatedly disrupted by looping. In the authors’ words: “by periodically breaking up self-associating chromatin domains, loop extrusion may provide an opportunity for factors in the nucleus to constantly sample these regions of the genome should they be required for future gene expression programs.” Perhaps related is evidence indicating that genes dependent on very distant enhancers (e.g., the mouse *Shh* gene) are particularly sensitive to cohesin loss.^26^ Churn may help to explain this preference, as the ability of a promoter to interact with remote enhancers is expected to be particularly vulnerable to failure of genome mixing, whereas interactions between nearby enhancer-promoter pairs may be less sensitive, initially at least.

Implicit in this view is that a primary function of cohesin is the *maintenance* of optimal gene expression. Loop extrusion is not continuous but occurs every so often, suggesting that its liberating effects take time to subside. This delay may explain the initially modest effect of cohesin ablation on transcription.

Higher-order structure of chromatin in the nucleus is often considered as a powerful means of choreographing genome activity. As well as the long-standing concepts of heterochromatin and euchromatin, there are genomic condensates whose functional significance is the subject of much current interest. These entities are perceived positively, as evolved ways of simplifying the management of large genomes by biasing macromolecular interactions in favor of those that are productive. Here it is proposed that compartments and condensates can also have negative connotations due to their potential to clog the genome and locally distort the concentration of protein factors. A potential example is provided by what are sometimes referred to as “transcription hubs,” which are dynamic local microenvironments thought to be important for gene expression.^27^ Hub composition and function vary widely. In the fruitfly, for example, only about one-third of Notch-containing hubs include mediator and RNA polymerase, and even in the presence of all the necessary transcription factors, active transcription is stochastic.^28^ Depletion of cohesin in this system causes the size of mediator-containing hubs to significantly increase, consistent with the possibility that loop extrusion normally disrupts these structures. While some hubs may indeed facilitate gene expression, it is possible that the tendency of transcription factors to coalesce is a mixed blessing and may even be deleterious unless restrained by periodic dispersal.

Central to the present argument is that cellular systems depend upon relatively uniform availability of chromosomal DNA to macromolecules to ensure effective regulation of the processes necessary for life. Cohesin can achieve this and in doing so help to ensure that the activity of protein factors is determined simply by their stoichiometry and binding affinities. Unless continual work is performed to overcome it, quasi-heterochromatic inaccessibility could be the eventual default state of much of the genome. An interesting corollary of this scenario is that local inhibition of cohesin activity may contribute to the initiation or maintenance of heterochromatization of parts of the genome. A speculative potential example is provided by the silencing of one X chromosome in female placental mammals to create facultative heterochromatin. As the inactive X chromosome lacks TADs, might there be a mechanistic link between *XIST* RNA induction and exclusion of active cohesin? Further investigation of this and other gene silencing phenomena may provide ways of testing the “churn-ase” model of cohesin function.
